# Supplementary material for: A high-resolution mRNA expression time course of embryonic development in zebrafish
Source: eLife. 2017 Nov 16;6:e30860. doi: 10.7554/eLife.30860 (PMC5690287; doi:10.7554/eLife.30860)
Supplement: Supplementary file 6. [file elife-30860-supp6.zip › biolayout-clusters-files/Cluster254-genes.html]

Cluster254


# Cluster254: Genes

| Ensembl ID | Gene Name | Chr | Start | End | Biotype |
| --- | --- | --- | --- | --- | --- |
| ENSDARG00000102032 | CABZ01063601.1 | KN150172.1 | 5708 | 10807 | protein\_coding |
| ENSDARG00000032098 | c8g | 24 | 16886710 | 16895219 | protein\_coding |
| ENSDARG00000078918 | comtd1 | 13 | 17541535 | 17554353 | protein\_coding |
| ENSDARG00000036171 | rnasel3 | 14 | 38313986 | 38316149 | protein\_coding |
| ENSDARG00000009401 | vcanb | 10 | 43360298 | 43434580 | protein\_coding |
| ENSDARG00000099200 | zgc:123103 | 19 | 42978262 | 42981901 | protein\_coding |
